# *TSC2* epigenetic defect in primary LAM cells. Evidence of an anchorage-independent survival

**DOI:** 10.1111/jcmm.12237

**Published:** 2014-03-07

**Authors:** Elena Lesma, Silvia Ancona, Silvia M Sirchia, Emanuela Orpianesi, Vera Grande, Patrizia Colapietro, Eloisa Chiaramonte, Anna Maria Di Giulio, Alfredo Gorio

**Affiliations:** a Laboratory of Pharmacology, Dept. of Health Sciences, Università degli Studi di MilanoMilano, Italy; b Laboratory of Medical Genetics, Dept. of Health Sciences, Università degli Studi di MilanoMilano, Italy

**Keywords:** tuberous sclerosis complex, lymphangioleiomyomatosis, epigenetic modification, mesenchymal transition

## Abstract

Tuberous sclerosis complex (TSC) is caused by mutations in *TSC1* or *TSC2* genes. Lymphangioleiomyomatosis (LAM) can be sporadic or associated with TSC and is characterized by widespread pulmonary proliferation of abnormal α-smooth muscle (ASM)-like cells. We investigated the features of ASM cells isolated from chylous thorax of a patient affected by LAM associated with TSC, named LAM/TSC cells, bearing a germline *TSC2* mutation and an epigenetic defect causing the absence of tuberin. Proliferation of LAM/TSC cells is epidermal growth factor (EGF)-dependent and blockade of EGF receptor causes cell death as we previously showed in cells lacking tuberin. LAM/TSC cells spontaneously detach probably for the inactivation of the focal adhesion kinase (FAK)/Akt/mTOR pathway and display the ability to survive independently from adhesion. Non-adherent LAM/TSC cells show an extremely low proliferation rate consistent with tumour stem-cell characteristics. Moreover, LAM/TSC cells bear characteristics of stemness and secrete high amount of interleukin (IL)-6 and IL-8. Anti-EGF receptor antibodies and rapamycin affect proliferation and viability of non-adherent cells. In conclusion, the understanding of LAM/TSC cell features is important in the assessment of cell invasiveness in LAM and TSC and should provide a useful model to test therapeutic approaches aimed at controlling their migratory ability.

## Introduction

Tuberous sclerosis complex (TSC) is an autosomal dominant disorder caused by inactivating mutations in *TSC1* or *TSC2* genes encoding hamartin and tuberin respectively [1–3]. The outcome of such genetic alterations is a multisystem disorder exhibiting a wide range of manifestations characterized by tumour-like lesions called hamartomas in various organs and pulmonary lymphangioleiomyomatosis (LAM) that may occur in association with TSC or sporadically [4,5]. Lymphangioleiomyomatosis is characterized by alveolar smooth muscle cell proliferation, and cystic destruction of lung parenchyma causing recurrent pneumothorax, dyspnoea and respiratory failure [6]. Identical *TSC2* mutations and loss of heterozygosity (LOH) patterns were found in LAM cells from lung nodules, angiomyolipomas (AMLs) and lymph nodes of the same sporadic LAM patient, suggesting that the two diseases share a common genetic origin; this is also consistent with metastatic spread among organs [7,8]. Furthermore, LAM cells were identified in donor lungs after transplantation and could be isolated from blood, urine and chylous effusion of patients with LAM [8,9]. Such behaviour of LAM cells with respect to their infiltrative growth pattern, metastatic potential and altered cell differentiation is reminiscent of cells undergoing epithelial-to-mesenchymal transition (EMT) [10].

The focal and variable nature of the hamartomas seen in TSC have long suggested that these tumours may develop following the two-hit model originally proposed for retinoblastoma by Knudson [11]. Loss of heterozygosity in *TSC1* or *TSC2* has been documented in LAM cells, in AMLs, and purified AML cells, in cardiac rhabdomyomas of patients, but it has only rarely been found in cerebral cortical tubers and skin lesions [12,13]. The inability to find a second somatic event in TSC lesions has been attributed to either different genetic and epigenetic alterations in *TSC* genes or cell heterogeneity in TSC hamartomas [14,15]. The absence of tuberin in smooth muscle-like cells from AML of a TSC2 patient caused by methylation of the *TSC2* promoter was recently described [16]. DNA methylation is an epigenetic change that induces chromatin modifications and repression of transcription *via* a methyl CpG binding proteins, and recruitment of a co-repressor complexes [17,18].

Here, from chylous effusion of a LAM/TSC patient, we report the isolation and characterization of a homogenous population of α-smooth muscle-like (ASM) cells with absence of tuberin for a *TSC2* mutation of one allele and an epigenetic alteration of the second allele. The proliferation of these cells was epidermal growth factor (EGF)-dependent and the blockade of EGF receptor (EGFR) caused cell death as we previously reported for tuberin null cells [16,19]. We studied the LAM/TSC cells’ ability to survive independently from the anchorage and to switch from adherent to a non-adherent status. Rapamycin and anti-EGFR antibodies caused reduction in cell growth and decreased anchorage-dependent survival. LAM/TSC cells secrete high amount of interleukin (IL)-6 and IL-8, cytokines with a crucial functional role in a variety of cancer cells [20].

## Materials and methods

### Cell cultures, treatments and proliferation assay

Chylous was obtained from a patient affected by LAM associated with TSC who had given her informed consent according to the Declaration of Helsinki. The study was approved by the Institutional Review Board of Milan*s San Paolo Hospital. Chylous was centrifuged and pellet repeatedly washed in PBS. Pellet was resuspended in Type II complete medium (50/50 mixture of DMEM/Ham F12; Euroclone, Paignton, United Kingdom) supplemented with 2.5 μg/ml hydrocortisone (Sigma-Aldrich, St. Louis, MO, USA), 10 ng/ml EGF (Sigma-Aldrich), 8.6 ng/ml sodium selenite (Sigma-Aldrich), 25 μg/ml insulin (Sigma-Aldrich), 10 μg/ml transferrin (Sigma-Aldrich), 0.445 μg/ml ferrous sulphate (Sigma-Aldrich) and 15% foetal bovine serum (FBS) (Euroclone) [19,21]. LAM/TSC cells were used between passage 5 and 15. TSC2^−/−^ and TSC2^−/meth^ ASM cells were isolated from AMLs of a female and male TSC2 patients respectively [16,19]. TSC2^−/−^ ASM cells are characterized by a germline *TSC2* exon 18 mutation consisting of a base pair change in amino acid 698 from a lysine to a stop codon (K698X) and a deletion (LOH) of the *TSC2* second allele [19]. TSC2^−/meth^ ASM cells are characterized by a germline *TSC2* intron 8-exon nine junction mutation (c.867-2A>G) and an epigenetic silencing of the second allele caused by promoter methylation [16]. TSC2^−/−^ and TSC2^−/meth^ ASM cells were grown in Type II complete medium with 15% FBS. Human aortic smooth muscle cells (HASMCs; Invitrogen, Carlsbad, CA, USA) were maintained in F12 medium with 10% FBS. COS7 (fibroblast-like cells; ATCC, Manassas, VA, USA), MCF7 (breast cancer cells; ATCC) and human mammary epithelial cells (HMECs; ATCC) were maintained in DMEM containing 10% FBS.

Proliferation was assayed in a Neubauer chamber in the presence or absence of EGF (10 ng/ml), after incubation with anti-EGFR antibody Ab3 (clone 225; Calbiochem, Darmstadt, Germany) or anti-EGFR antibody Ab5 (EGFR.1; Calbiochem) at the concentration of 5 μg/ml, and rapamycin at the concentration of 1 or 5 ng/ml (Rapamune-Sirolimus, Wyeth Europa, United Kingdom). Trichostatin A (Sigma-Aldrich) was incubated for 72 hrs (1 ng/μl), 5-azacytidine (Sigma-Aldrich) for 96 hrs (0.24 ng/μl) and PF-537228 (PF228; Sigma-Aldrich) for 8 hrs (0.5 and 5 ng/μl).

### Cell immunofluorescence microscopy

The cells, cultured on glass slides and permeabilized with Cytoskelfix (Cytoskeleton, Denver, CO, USA), were incubated overnight at 4°C with the primary antibodies (Table 1). Samples were incubated for 3 hrs at room temperature with Alexa 488- or Alexa 555-conjugated secondary antibody (Invitrogen). The fluorescence of the cells was analysed by using a laser scanning confocal microscope (LEICA DMIRE2, Wetzlar, Germany). Nuclei were stained with DAPI (2 μg/ml; Sigma-Aldrich).

**Table 1:** List and dilution of primary antibodies

Primary antibodies	Dilution	Supplier
α-actin	1:100	Sigma-Aldrich
HMB45	1:100	DAKO
CD44v6	1:100	Invitrogen
Hamartin	1:100	Santa Cruz
Tuberin (carboxy-terminus)	1:1000	Cell Signaling
Tuberin (amino-terminus)	1:1000	Cell Signaling
Phospho-tuberin (Thr1462)	1:1000	Cell Signaling
E-Cadherin	1:50	BD
Phospho-S6 ribosomal protein (Ser235/236)	1:1000	Cell Signaling
S6 ribosomal protein	1:1000	Cell Signaling
Phospho-Erk (Thr202/Tyr204)	1:1000	Cell Signaling
Erk	1.1000	Cell Signaling
Phospho-Akt (Ser473)	1:1000	Cell Signaling
Phospho-mTOR (Ser2448)	1:1000	Cell Signaling
Phospho-mTOR (Ser2481)	1:1000	Cell Signaling
Vimentin	1:1000	Cell Signaling
SNAIL	1:1000	Cell Signaling
Phospho-FAK (Tyr397)	1:100	Santa Cruz
Cdk4	1:100	Santa Cruz
Cyclin D1	1:100	Santa Cruz
PCNA	1:250	Chemicon
β-actin	1:1000	Sigma-Aldrich

DAKO, Carpinteria, CA, USA; Santa Cruz Biotechnology, Santa Cruz, CA, USA; Cell Signaling, Beverly, CA, USA; Chemicon, Temecula, CA, USA; BD Bioscience, San Josè, CA, USA.

### Western blotting

Cells were lysed in lysis buffer (1.9 mg/ml ethylenediaminetetraacetic acid, 8.2 mg/ml deoxycholic acid, 3% SDS), electrophoretically run on a 10% sodium SDS-PAGE and transferred to nitrocellulose membranes (Amersham, Arlington Heights, IL, USA). After being blocked at room temperature for 1 hr with 5% dry milk (Merck Millipore, Darmstadt, Germany), membranes were incubated overnight at 4°C with primary antibodies (Table 1). The membranes were incubated for 1 hr with secondary antibodies (1:10,000) horseradish peroxidase conjugated (Pierce, Rockford, IL, USA). The reaction was revealed by using the SuperSignal West Pico chemiluminescent substrate (Pierce). Images were acquired on a Kodak image station 440 CF. Densitometric analysis was performed by using Kodak MJ project program (Kodak, Milan, Italy) and results were expressed as the mean value from at least three independent experiments relatively to β-actin levels.

### Mutation study and LOH analysis

DNAs were extracted from cultured cells by using the Wizard Genomic DNA purification kit (Promega, Madison, WI, USA). All of the exons and intron-exons junctions of *TSC1* and *TSC2* from the genomic DNAs were amplified by means of standard PCR and described primers [22]. The sequencing reactions were performed as previously reported by Lesma *et al*. [16]. Loss of heterozygosity was analysed as previously described in Lesma *et al*. [16].

### Pyrosequencing analysis

To quantify the methylation levels of *TSC2* promoter, pyrosequencing technology was used to analyse the *TSC2* CpG island (CpG 94, chr16:2097466-2098365, UCSC Genome Browser). DNA conversion and PCR reaction were carried out as previously described [23] by using the primers and conditions described in Table 2. The analysed sequences are shown in Figure 2. Pyrosequencing reactions were performed as previously described in Sirchia *et al*., [24]. To assess the methylation pattern in normal condition, we analysed different normal DNA from peripheral blood lymphocytes.

**Table 2:** Conditions and primers sequences for pyrosequencing assay

Method	Primer sequences	Annealing	Nr of CpG investigated
PCR 1	F1 5′-TTYGTTAGAGGGYGGTATAGAAT-3′	52°C	
	R1 5′-bio ACACTACRAAATCCRCCTCTC-3′		
Pyrosequencing 1	S1 5′-ATYGGAAGTGYGGGT-3′		10
PCR 2	F2 5′-GGAAGYGTTTTTTTGTTTGG-3′	55°C	
	R2 5′-bio CCRCRCCRTAACTAACTAAAACRTA-3′		
Pyrosequencing 2 and 3	S2 5′-GGYGAAAGGGGGTAG-3′		6
	S3 5′-TGTAATTTTTGGGAAAAAG-3′		8
PCR 3	F3 5′-TAYGTTTTAGTTAYGGYGYGG-3′	54°C	
	R3 5′-bio ATAATTCTATACCRCCCTCTA-3′		
Pyrosequencing 4	S4 5′-GGTTAGTATTTT-3′		4

### Chromatin immunoprecipitation (ChIP) assay

Chromatin immunoprecipitation assay was performed with the ChIP assay kit (Merck Millipore) by using anti-AcH3, anti-H3 lysine 4 trimethylation (H3TrimK4) and anti-H3 lysine 9 trimethylation (H3TrimK9) antibodies (Merck Millipore), according to the manufacturers’ instructions. Chromatin was immunoprecipitated from 1 to 3 × 10^6^ cells, to work in saturating conditions for each antibody. Chromatin fractions obtained by immunoprecipitation were purified by using QIAquick PCR purification kit (Qiagen, Hilden, Germany), following the manufacturer*s instructions. Quantification of the TSC2 promoter region in chromatin fractions was performed by TaqMan-Real Time PCR by using primers F: 5′-GTCAGCTGGGCTGTAGTTGAGTT-3′ and R: 5′-TCTGGTGGGCATAACCTTTCC-3′ and TaqMan probe 6FAM- CCCAGGGAGTGTGGG. The TaqMan assay was designed by using Primer Express 3.0 Software (Applied Biosystems, Foster, CA, USA). All experiments were performed by using StepOne™ Real-Time PCR System with Fast protocol, using TaqMan® Fast Universal PCR Master Mix (2×), No AmpErase® UNG (Applied Biosystems) on StepOne™ Real-Time PCR System. The amount of TSC2 promoter was calculated by using the 2^−ΔΔCt^ method relative to INPUT sample (chromatin before immunoprecipitation). Background (negative control without antibody) was subtracted from all samples. To assess the normal chromatin pattern, we analysed HMECs.

### Trypan blue exclusion assay

Quantification of viable cells was performed by incubating the cells in trypan blue staining solution (1:25; Sigma-Aldrich) for 8 min. at room temperature. Cells were counted in Neubauer chamber. Results represent the number of viable cells over the total number of cells counted.

### Anchorage-independent growth assay

10^5^ cells of adherent LAM/TSC cells and HASMC were plated. Plates were prepared as follows: 2% agarose solution was mixed with an equal volume of Type II Complete Medium and poured onto 35 mm petri dish, forming an agarose base. Then, a 0.6% agarose solution was mixed with an equal volume of Type II Complete Medium containing the cell suspension producing a 0.3% agarose solution. After solidification, culture medium was added to each plate. Two weeks after plating, colonies were visualized by using 0.4% iodonitrotetrazolium solution (Sigma-Aldrich). Colonies were counted by light microscopy. The results represent the average number of colonies per 35-mm plate.

### Quantitative real-time PCR

RNA was extracted by using Trizol reagent (Invitrogen). cDNA was then synthesized by reverse transcription of 1 μg of total RNA by using the SuperScript VILO cDNA synthesis kit (Invitrogen). Quantitative real-time PCR was performed by using MJ Opticon PCR analyser (MJ Research Inc., Waltham, MA, USA). The resulting cDNA was analysed by using the Taqman system for quantitative evaluation of specific transcripts according to the manufacturer*s instructions (Applied Biosystems). All mRNA expression data were normalized to 18S expression (Eukaryotic 18s rRNA endogenous Control) in the corresponding sample. For the analysis, primers for proliferating cellular nuclear antigen (PCNA; Hs 00696862_m1) were obtained from Applied Biosystems ‘Assays on Demand’ system (Applied Biosystem). PCR reaction involved one cycle of 50°C for 2 min., one cycle of 95°C for 10 min. and 40 cycles of 95°C for 15 sec. and 60°C for 1 min. The results were calculated by Ct (2^−ΔΔCt^) method.

### Flow cytometric analysis of the cell cycle and S6 activation

Cells (∼1 × 10^6^), grown overnight without serum, were collected by centrifugation, fixed with 70% ethanol overnight at −20°C, washed in PBS and resuspended in 1 ml of staining solution (10 μg/ml propidium iodide and 200 μg/ml RNase A in PBS with 0.04% Nonidet-P40). Samples were incubated overnight at 4°C in the dark. For the quantification of S6 phosphorylation, cells (∼2 × 10^6^) were fixed with fixation buffer (BD, Becton Dickinson Italia, Buccinasco, Milan, Italy) for 1 hr at 4°C, washed and permeabilized with Perm/Wash buffer I (BD) for 1 hr at room temperature, incubated with Alexa Fluor 647 anti-phospho-S6 (S235/S236; BD) for 1 hr at room temperature. Samples were analysed with the flow cytometer Cytomics FC500 and analysis of cell cycle phases was performed with the CXP 2.2 software (Beckman Coulter, Milan, Italy).

### ELISA

Quantification of IL-1α, IL-6, IL-1β and IL-8 in supernatant from LAM/TSC cells was accomplished by Elisa in accordance with the manufacturer*s instructions (Invitrogen). LAM/TSC cells were incubated with 5-azacytidine (0.24 ng/μl), rapamycin (1 ng/ml) or anti-EGFR Ab3 (5 μg/ml), metilprednisolone (3.5 μg/μl; Sigma-Aldrich), repertaxin (0.43 μg/μl; Sigma-Aldrich) or cycloheximide (10 μg/ml; Sigma-Aldrich).

### Statistical analysis

Data are expressed as mean ± SD of n independent experiments. Comparisons of three or more groups were performed by anova, followed by Bonferroni*s test. Comparison between two groups was performed by Student*s *t*-test. *P* < 0.05 was considered statistically significant.

## Results

### Proliferation of LAM/TSC cells is EGF-dependent and anti-EGFR antibody causes cell death

**Fig. 1:**
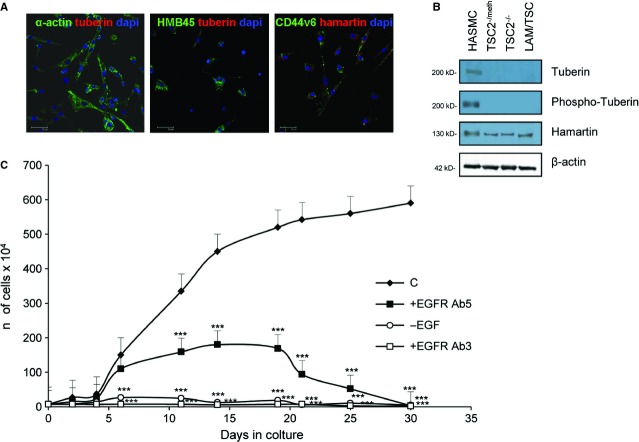
Characterization of lymphangioleiomyomatosis/tuberous sclerosis complex (LAM/TSC) cells. (**A**) Cells are positive to α-actin (green labelling) and negative to tuberin antibodies (red labelling; left panel), positive to HMB45 (green labelling) and negative to tuberin antibodies (red labelling; middle panel), positive to CD44v6 (green labelling) and hamartin antibodies (red labelling; right panel). Nuclei are stained with DAPI (blue); scale bars: 50 μm. (**B**) Representative immunoblots show tuberin and its phosphorylated form, and hamartin. β-actin was evaluated as loading control. (**C**) Proliferation of LAM/TSC cells was evaluated up to 30 days in the presence of epidermal growth factor (EGF; 10 ng/ml) with EGF receptor (EGFR) Ab3 (5 μg/ml), EGFR Ab5 (5 μg/ml) and without EGF. Error bars represent SD for four independent experiments.***P < 0.001 *versus* control (anova with Bonferroni*s test).

From the chylous effusion of a patient affected by LAM associated with TSC, we isolated a homogenous population (LAM/TSC cells) positive to α-actin antibody, marker of smooth muscle cells, hamartin, and the specific markers for TSC and LAM cells, HMB45 and CD44v6 antibodies (Fig. 1A). Tuberin and its Thr1462-phosphorylated form were not expressed in LAM/TSC cells (Fig. 1A and B). TSC2^−/meth^ and TSC2^−/−^ ASM cells were used as negative control, for their absence of tuberin, and HASMCs as positive control. Proliferation of LAM/TSC cells was EGF-dependent (Fig. 1C) as we previously reported also for TSC2^−/−^ and TSC2^−/meth^ ASM cells [16,19,25]. The exposure to two different anti-EGFR antibodies affected LAM/TSC cell proliferation and caused their progressive death. The antibody directed to the ligand binding site (clone 225: EGFR Ab3) quickly blocked proliferation causing cell death within 2 weeks. Differently, the antibody directed to the cell surface domain (clone EGFR.1: EGFR Ab5) affected proliferation between 5 and 10 days after exposure causing progressive cell death after 20 days of incubation.

### LAM/TSC cells do not express tuberin for an epigenetic modification

**Fig. 2:**
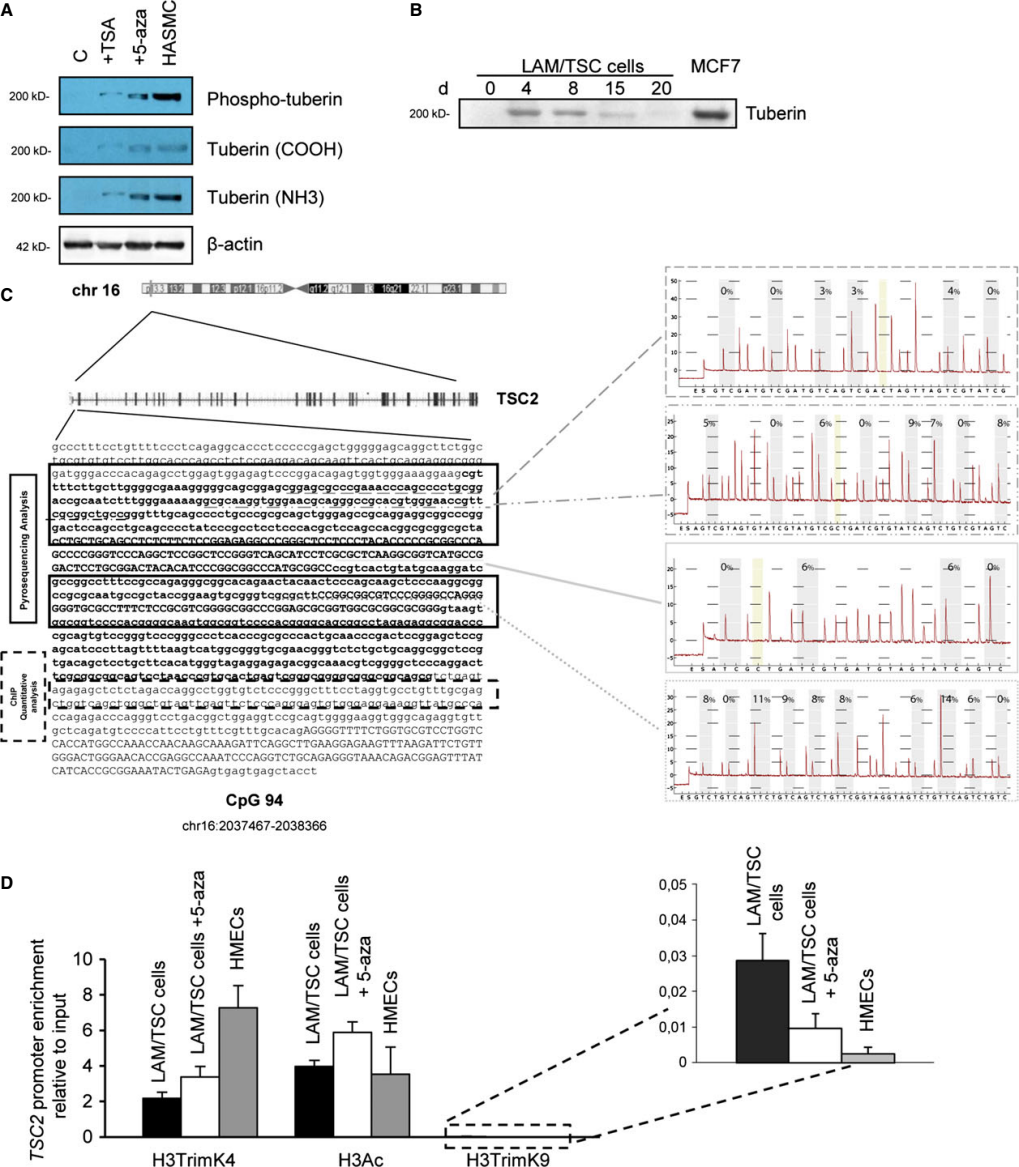
Lymphangioleiomyomatosi/tuberous sclerosis complex (LAM/TSC) cells do not express tuberin for an epigenetic modification. (**A**) Incubation with trichostatin A (TSA) or 5-azacytidine (5-aza) promoted the expression of tuberin and phospho-tuberin in LAM/TSC cells, as evaluated by western blotting. HASMCs were used as control. (**B**) Tuberin expression was evaluated after incubation with 5-aza for 20 days. MCF7 cells were used as control. (**C**) Pyrosequencing analysis for CpG islands was performed in four regions without finding methylated pattern. (**D**) *TSC2* promoter chromatin status was analysed by ChIP assay to study repressed chromatin (H3TrimK9 antibody) and open chromatin (H3TrimK4 and H3Ac antibodies) in LAM/TSC cells and after incubation with 5-azacytidine. Human mammary epithelial cells were used as control. Error bars represent the SD for *n* = 3.

Lymphangioleiomyomatosis/TSC cells bear a *TSC2* germline mutation on exon 21 (R750X-2251 C>T). Loss of heterozygosity was not detectable by using a panel of microsatellite markers close to the *TSC2* locus on chromosome 16p13.3 (data not shown). As tuberin is undetectable in LAM/TSC cells, we hypothesized that the presence of an epigenetic defect underlying the silencing of the *TSC2* wild-type allele. It is well known that H3 lysine 9 methylation and CpG methylation may occur in heterochromatic regions and are associated with silencing of gene expression, whereas the acetylation of histones H3 and H4, and H3 lysine 4 methylation correlate with euchromatin and transcriptionally active genes [26]. Treatment of LAM/TSC cells with trichostatin A, a histone deacetylase inhibitor, or 5-azacytidine that inhibits CpG DNA methylation led to the expression of tuberin as consequence of the chromatin remodelling (Fig. 2A). The positive effect of 5-azacytidine on tuberin expression can be observed for up to 2 weeks following the incubation with the drugs (Fig. 2B). To assess the epigenetic status of the *TSC2* gene, by pyrosequencing assay, we analysed DNA methylation of four regions that are considered the exemplification of the entire *TSC2* CpG islands (Fig. 2C). In this region, we previously observed methylation associated with *TSC2* silencing in cells derived from an AML of a TSC2 patient [16]. Both LAM/TSC cells and normal control show unmethylated patterns in all the sequences studied (Fig. 2C). Nevertheless, the presence of tuberin appears to be related to a *TSC2* epigenetic control, given that the chromatin-remodelling treatments restore tuberin expression (Fig. 2A and B). These findings suggest that the methylation of *TSC2* CpG islands is not a primary event in the *TSC2* epigenetic regulation of LAM/TSC cells. Therefore, we evaluated the *TSC2* promoter chromatin status by ChIP assay. We found an increase of repressed chromatin signature (H3TrimK9) together with a reduction of open chromatin markers (H3TrimK4 and H3Ac) in LAM/TSC cells compared with HMECs (Fig. 2D). The *TSC2* promoter chromatin structure in LAM/TSC cells is consistent with tuberin-silenced status. After incubation with 5-azacytidine, we observed a reduction of H3TrimK9 and an increase of H3TrimK4 and H3Ac, indicating the opening of the *TSC2* chromatin structure leading to expression of tuberin (Fig. 2D).

### Rapamycin efficiently blocks proliferation of LAM/TSC cells

**Fig. 3:**
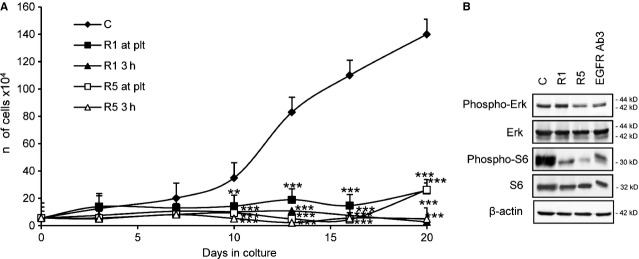
Rapamycin inhibits proliferation of lymphangioleiomyomatosis/tuberous sclerosis complex (LAM/TSC) cells. (**A**) Proliferation was evaluated after incubation with rapamycin (R) at plating time (at plt) at the concentration of 1 ng/ml (1) or 5 ng/ml (5) or 3 hrs after plating (3 h). Error bars represent SD for four independent experiments. Error bars represent SD for four independent experiments.**P < 0.01 and ***P < 0.001 *versus* control (anova followed by Bonferroni*s test).(**B**). Phospho-S6 and S6, phospho-Erk and Erk levels were evaluated following incubation with rapamycin and EGF receptor Ab3 for 6 days 3 hrs after plating. β-actin was evaluated as loading control.

Rapamycin significantly reduced proliferation either when it was added at the medium at plating time or 3 hrs after plating, when the cells were attached to the dish (Fig. 3A). These data differ from that observed with TSC2^−/−^ and TSC2^−/meth^ ASM cells, where rapamycin was mostly effective in reducing the proliferation only when administered at plating time [19,27]. Hyperphosphorylation of both p70S6K and its ribosomal protein S6 substrate has been observed previously in genetically modified cells lacking tuberin [28,29]. Incubation with anti-EGFR antibody and rapamycin reduced S6 phosphorylation without affecting protein expression as quantitatively demonstrated by densitometric analysis (Fig. 3B and Fig. S1). Erk phosphorylation was also affected by the two agents, although rapamycin was effective only at the higher dose (Fig. 3B and Fig. S1). Erk expression was unchanged (Fig. 3B and Fig. S1).

### LAM/TSC cells spontaneously detach and grow independently from anchorage

**Fig. 4:**
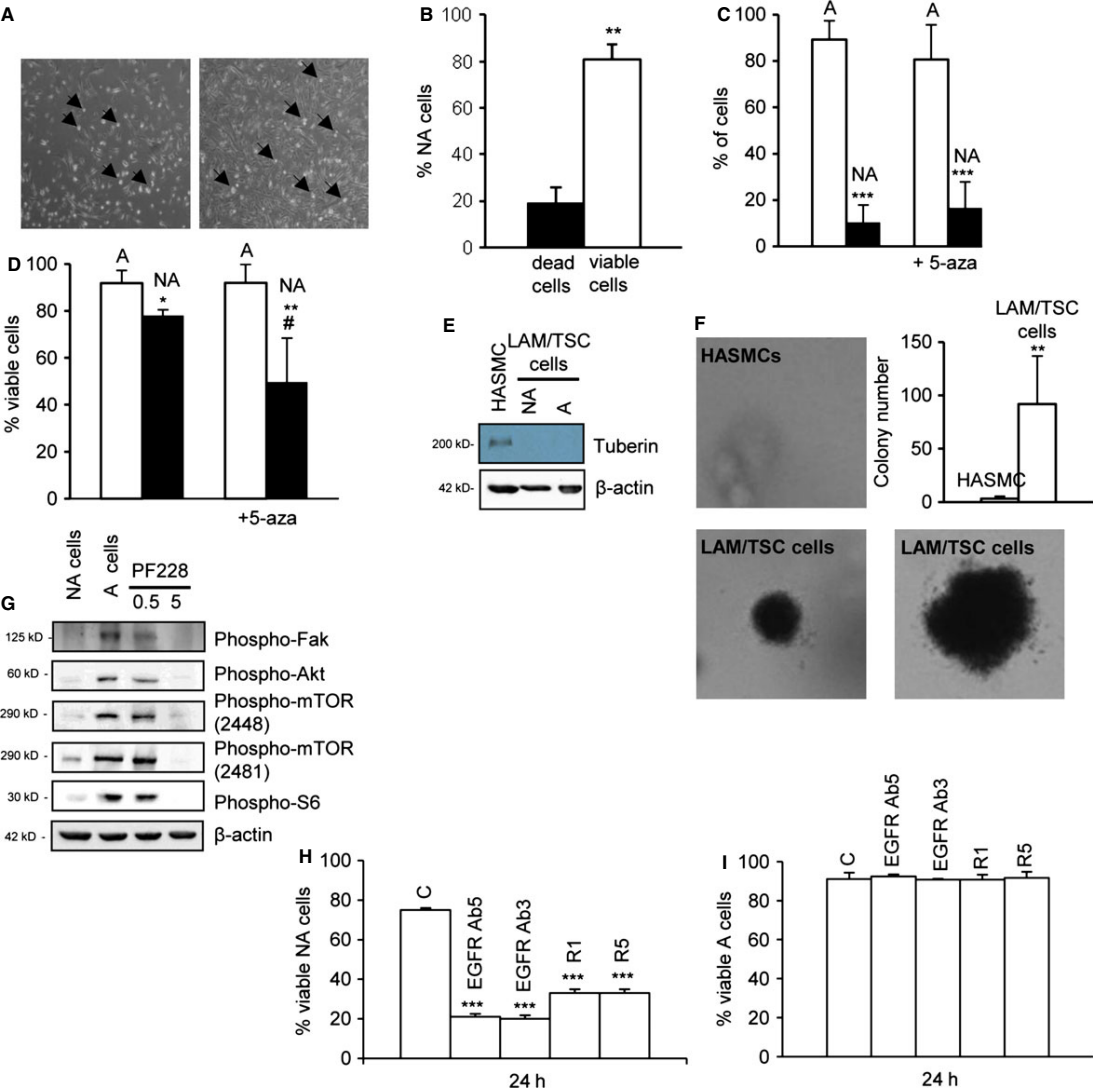
Lymphangioleiomyomatosis/tuberous sclerosis complex (LAM/TSC) cells survive in adherent (A) and non-adherent (NA) status independently from anchorage. (**A**) Representative microscopy images with LAM/TSC cells that detach (arrows) from the dish in subconfluent and confluent condition (left and right panel, respectively). (**B**) Viability of NA LAM/TSC cells, analysed by Trypan blue assay, is expressed as percentage of dead or viable NA LAM/TSC cells. Error bars represent the SD for three independent experiments. Unpaired Student*s *t*-test p-values: **P < 0.01 *versus* dead cells. (**C**) The percentage of NA and A LAM/TSC cells was evaluated in control cells and after incubation with 5-azacytidine (5-aza). Error bars represent the SD for three independent experiments. Unpaired Student*s *t*-test p-values: ***P < 0.001 *versus* A cells. (**D**) Viability of A and NA LAM/TSC cells was analysed in control cells and after incubation with 5-aza. Error bars represent the SD for three independent experiments. Unpaired Student*s *t*-test p-values: *P < 0.05 and **P < 0.01 *versus* A LAM/TSC cells; #*P* < 0.05 *versus* control NA LAM/TSC cells. (**E**) Evaluation of tuberin expression in A, NA LAM/TSC cells and human aortic smooth muscle cells (HASMCs) used as control. β-actin is the loading control. (**F**) Representative colony formation in soft agar of LAM/TSC cells and HASMCs, used as control, assessed 2 weeks after plating to test anchorage-independent survival (magnification 20 × ). Graph indicates number of LAM/TSC cell colonies compared with colonies of HASMCs. Error bars represent the SD for three independent experiments. Unpaired Student*s *t*-test p-values: **P < 0.01 *versus* HASMCs colonies. (**G**) Phospho-focal adhesion kinase, phospho-Akt, phospho-mTOR (Ser 2448), phospho-mTOR (Ser2481) and phospho-S6 in A and NA LAM/TSC cells and after incubation with PF228 (0.5 or 5 ng/μl). β-actin was evaluated as loading control. (**H**) The percentage of viable NA and (**I**) viable A cells was evaluated by trypan blue assay after 24 hrs of incubation with rapamycin (R) at the concentration of 1 (1) or 5 (5) ng/ml, and anti-EGF receptor (EGFR) antibodies (EGFR Ab3 and EGFR Ab5). Error bars represent the SD for three independent experiments. ***P < 0.001 *versus* control cells (anova followed by Bonferroni*s test).

It has been described that the absence of tuberin in renal epithelial tumour cell lines from Eker rats leads to reduction of both adhesion and formation of aggregates [30]. We observed that LAM/TSC cells can spontaneously detach from the sub-confluent monolayer and grow for some time in suspension, then re-attach to the plate and grow in monolayer again (Fig. 4A). The majority of the non-adherent LAM/TSC cells were viable (Fig. 4B). About 10% of LAM/TSC cells were floating after 24 hrs of culture (Fig. 4C), while the percentage was slightly higher (17%) after incubation with 5-azacytidine for 96 hrs (*ns versus* non-adherent control cells) likely because of a cytotoxic effect of the chromatin-remodelling agent. In fact, 5-azacytidine reduced significantly the viability of non-adherent cells compared with non-adherent untreated cells without any effect on the viability of adherent cells (Fig. 4D). Tuberin was not expressed either in adherent and non-adherent LAM/TSC cells (Fig. 4E). LAM/TSC cells survived and grew when cultured in soft agar, where they formed colonies, indicating their loss of contact inhibition and anchorage-independent survival (Fig. 4F). Differently, HASMCs failed to form colonies in soft agar, showing an adhesion-dependent growth.

Focal adhesion kinase (FAK) is a non-receptor cytosolic kinase concentrated in focal contacts and probably involved in regulation of migration and survival mediated by contact with extracellular matrix protein [31]. Phosphorylation of FAK was reduced in non-adherent LAM/TSC cells compared with adherent cells (Fig. 4G). A similar result was observed for Akt, S6 and mammalian target of rapamycin (mTOR) phosphorylation. The low S6 phosphorylation of non-adherent cells was confirmed also by flow cytometric analysis (13.31 ± 3.95%; *P* < 0.01 *versus* adherent cells), while positivity was high (82.3 ± 2.47%) in adherent cells. To evaluate if the loss of contact in non-adherent LAM/TSC cells might be the event causing the inhibition of FAK/Akt pathway, adherent LAM/TSC cells were incubated with PF228, a FAK inhibitor. FAK phosphorylation was significantly reduced by 5 ng/μl of PF228 and slightly affected at the dose of 0.5 ng/μl (Fig. 4G and Fig. S2). FAK is an upstream regulator of Akt signalling pathway and FAK-Akt interaction is particularly critical for metastatic adhesion [32]. In LAM/TSC cells, FAK inhibition caused the reduction of Akt phosphorylation, which was followed by inhibition of phosphorylation and autophosphorylation of mTOR, and consequently by a strong reduction of S6 phosphorylation as it occurs in non-adherent cells (Fig. 4G). By densitometric analysis, PF228 (5 ng/μl) caused a statistically significant inhibition of the phosphorylation of Akt, mTOR and S6 compared with adherent control cells (Fig. S2). These data suggest that LAM/TSC cell detachment might control phosphoinositide 3-kinase (PI3K)/mTOR pathway through FAK. In cells that survive and proliferate attached to the dish, such as HASMCs, TSC2^−/−^ and TSC2^−/meth^ ASM cells, as expected, phosphorylation of FAK, Akt, mTOR and S6 was significantly reduced by PF228 (5 ng/μl), suggesting that LAM/TSC cells detach from the dish for the inhibition of FAK/Akt/mTOR pathway, but this event does not appear to be related to *TSC2* alterations (Fig. S3A and B). Rapamycin and anti-EGFR antibodies affected the viability of non-adherent LAM/TSC cells, while the drugs did not have any statistically significant effect in adherent cells (Fig. 4H and I).

### Replication of LAM/TSC cells is lower in non-adherent than in adherent condition

**Fig. 5:**
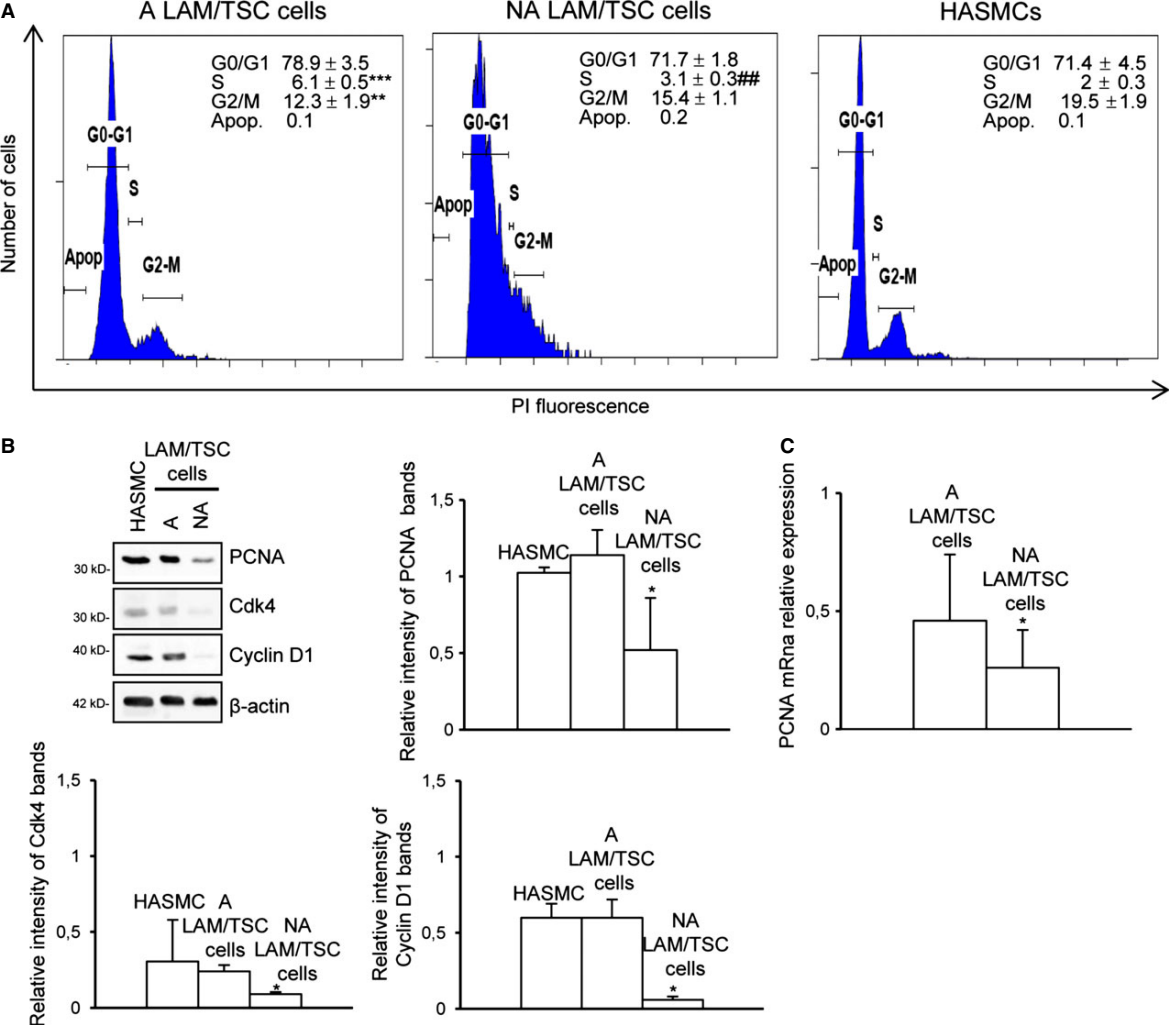
Proliferation of lymphangioleiomyomatosis/tuberous sclerosis complex (LAM/TSC) cells in non-adherent status and adherent condition. (**A**) Exemplificative linear fluorescence histograms of PI of different phases of cell cycle were analysed by flow cytometric analysis in human aortic smooth muscle cells (HASMCs,) non-adherent (NA) and adherent (A) LAM/TSC cells. Abscissa indicates relative intensity of PI fluorescence and ordinate relative number of cells. Data are expressed as mean ± SD of three independent experiments. **P < 0.01 and ***P < 0.001 *versus* HASMCs; ##*P* < 0.01 *versus* A LAM/TSC cells (anova followed by Bonferroni*s test). (**B**) Representative western blots show proliferating cellular nuclear antigen (PCNA), Cdk4 and cyclin D-1 expression in A and NA LAM/TSC using HASMCs as control. β-actin is the loading control. Relative intensity by densitometric analysis was evaluated relatively to β-actin levels. Error bars represent the SD for four experiments. *P < 0.05 *versus* HASMCs (anova with Bonferroni*s test). (**C**) PCNA mRna relative expression was analysed by RT-PCR in A and NA LAM/TSC cells considering HASMCs value as 1 unit. Error bars represent the SD for three experiments. Unpaired Student*s *t*-test p-values: *P < 0.05 *versus* HASMCs.

For reasons of the different extent of S6 phosphorylation in adherent and non-adherent cells, and the S6 role in the process of growth, we evaluated the proliferative status of LAM/TSC cells by flow cytometric analysis. We found that most adherent cells were in high replication condition with a higher DNA replication phase S compared with non-adherent LAM/TSC cells and HASMCs used as control (Fig. 5A). The floating cells were less in G0/G1 phase than adherent cells. The number of apoptotic cells in any group was low. To better analyse the replication of adherent and non-adherent LAM/TSC cells, we evaluated specific cyclin-dependent kinases (Cdks), such as Cdk 4, Cdk2 and Cdk6, which control the transition from G1 to the S phase of the cell cycle, by combining with their appropriate cyclin D [33]. Deregulation of Cdk4 and cyclin D1 is widespread in human cancer. The expression of Cdk4 and cyclin D1 is decreased in non-adherent LAM/TSC cells compared with the adherent cells (Fig. 5B). Moreover, we analysed PCNA, essential for DNA replication and repair of DNA errors, highly expressed during G1 and S-phases that decrease in G2 and M-phases [34]. Proliferating cellular nuclear antigen protein level and mRNA expression were significantly reduced in non-adherent LAM/TSC cells compared with adherent cells (Fig. 5B and C).

### LAM/TSC cells express EMT features

**Fig. 6:**
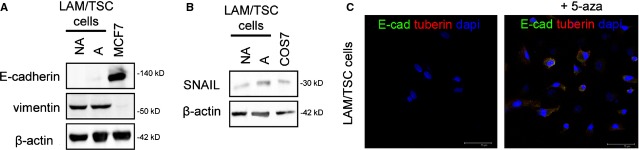
Lymphangioleiomyomatosis/tuberous sclerosis complex (LAM/TSC) cells express epithelial-to-mesenchymal transition marker. (**A**) Evaluation of E-cadherin and vimentin expression in non-adherent (NA) and adherent (A) LAM/TSC cells. MCF7 cells were used as control. β-actin is the loading control. (**B**) SNAIL is present in A and NA LAM/TSC cells such as in COS7 cells used as control. β-actin was the loading control. (**C**) Representative immunofluorescence microscopy images of E-cadherin staining (green labelling), tuberin expression (red labelling) in control LAM/TSC cells and after 5-azacytidine (5-aza) exposure. Nuclei were stained with DAPI (blue); scale bars: 75 μm.

One of the steps driving EMT is the repression of E-cadherin, resulting in the loss of cell–cell adhesion. According to Barnes *et al*. [30], the loss of tuberin in the Eker rat-derived cells leads to a significant reduction in membrane E-cadherin. Adherent and non-adherent LAM/TSC cells did not express E-cadherin, while vimentin, marker of mesenchymal cells, was detected (Fig. 6A). SNAIL, a transcription factor regulating E-cadherin expression and marker of epithelial mesenchymal transition, was identified by western blotting in LAM/TSC cells as it occurs in COS7 cells used as control (Fig. 6B). When tuberin expression was induced by exposure to 5-azacytidine, LAM/TSC cells were positively labelled by E-cadherin antibody in plasma membrane and cytoplasm (Fig. 6C). These observations support the concept that tuberin expression modulates the EMT features of LAM/TSC cells also in human TSC2 cells.

### LAM/TSC cells secrete IL-6 and IL-8

**Fig. 7:**
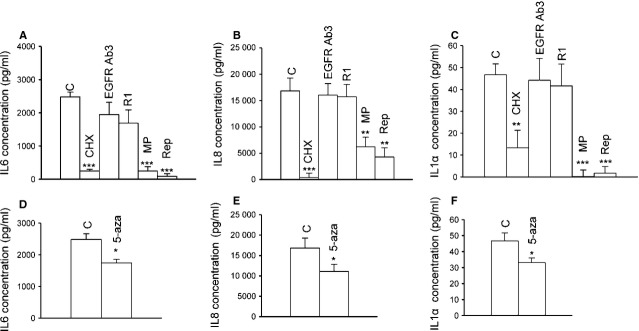
Lymphangioleiomyomatosis/tuberous sclerosis complex (LAM/TSC) cells secrete interleukins. (**A**) IL-6 (**B**) IL-8 and (**C**) IL-1α secretion were evaluated by Elisa in control LAM/TSC cells and after cycloheximide (CHX) overnight incubation. The effect of anti-EGF receptor (EGFR) antibody (EGFR Ab3), rapamycin (R1), methylprednisolone (MP) and repertaxin (Rep) after 48 hrs of incubation was evaluated. **P < 0.01 and ***P < 0.001 *versus* control LAM/TSC cells (anova followed by Bonferroni*s test). (**D**) IL-6 (**E**) IL-8 and (**F**) IL-1α release was evaluated after 5-azacytidine (5-aza) incubation. Error bars represent the SD for three experiments. Unpaired Student*s *t*-test p-values: *P < 0.05 *versus* control LAM/TSC cells.

Tumour cells migrate from mouse primary tumours through a process of EMT, and this process is dependent on an inflammatory microenvironment [20]. Likewise, IL-6 promotes EMT in breast cancer cells, and SNAIL can induce IL-6 expression in keratinocytes [35,36]. Interleukin-8 and IL-6 production is regulated by PI3K and mitogen-activated protein kinase pathways, which are also involved in TSC cell deregulated functions [37–39]. Interleukin-1α, IL-6 and IL-8 were secreted by LAM/TSC cells as the inhibition of protein biosynthesis by incubation with cycloheximide prevented their secretion (Fig. 7A–C). The release of the interleukins was, however, not significantly affected by rapamycin or anti-EGFR antibody exposure. Interleukin-6, IL-8 and IL-1α secretion was inhibited by the incubation with 5-azacytidine leading to tuberin expression (Fig. 7D–F). As expected, methylprednisolone and repertaxin, an antagonist of IL-8 receptor, effectively reduced the release of interleukins [40] (Fig. 7A–C). Interleukin-1β was not detectable.

## Discussion

The loss of TSC2 function leads to a spectrum of diseases that underlie abnormalities in cell growth, proliferation, differentiation and migration [41]. Despite the extensive biochemical and molecular characterization of TSC1/TSC2 pathway, the mechanisms linking these findings to the phenotype remain, however, quite not understood. Tuberous sclerosis complex is a tumour-suppressor gene syndrome associated with benign and, more rarely, malignant tumours. In this study, we describe the characterization of ASM cells derived from chylous effusion of a patient affected by LAM associated with TSC. LAM/TSC cells are positive for the LAM and TSC markers, CD44v6 and HMB45 antibody. The reactivity of LAM/TSC cells with HMB45 antibody was heterogeneous, as previously reported for other TSC and LAM cells [16,27,42]. The difference in HMB45 positivity has been related to the expression of tuberin and to the progression of LAM. LAM/TSC cells bear a germline mutation in exon 21 of *TSC2* and do not express tuberin because of an epigenetic silencing of the *TSC2* second allele that confirms our previously published evidence that an epigenetic alteration of *TSC2* can cause the loss of tuberin in TSC cells [16]. The process of inactivation of tumour suppressor genes by DNA methylation at CpG dinucleotides is common in human cancers and results in the loss of gene functions [18,43]. Recent developments in cancer research have shown that epigenome modifications are significantly associated with the development and progression of tumours. In our LAM/TSC cells, no methylated CpGs were detected, although the entire *TSC2* CpG island had been screened for DNA methylation by pyrosequencing analysis. However, we found histone modifications characterizing closed chromatin domains in the *TSC2* control region and tuberin expression after chromatin-remodelling agent treatment, thus the loss of tuberin is associated with epigenetic silencing of the *TSC2* gene, as observed in TSC2^−/meth^ ASM cells. The epigenetic regulation of gene expression is a very complex and multifactorial process in which a different combination of histone modifications and DNA methylation can be observed far or near inactivated genes [44]. In the light of this considerations, our findings in TSC2^−/meth^ ASM cells [16] and LAM/TSC cells indicate that different epigenetic signatures related to closed chromatin conformation can affect *TSC2* and may be crucial in the development of TSC lesions because they lead to the absence of tuberin expression as it is caused by the deletion of the wild-type allele in TSC2^−/−^ ASM cells. A better comprehension of the aberrant epigenetic events occurring in LAM/TSC cells may be useful in the development of more effective therapeutic interventions also based on chromatin-remodelling agents aimed at the epigenetic silencing relief of the *TSC2* [16,45].

The LAM/TSC cells display the physiological ability to survive in adherent and non-adherent status with no expression of tuberin in both conditions. The process is apparently highly correlated with changes in FAK activity that is high in adherent cells, as expected for cells preparing to migrate, and low in non-adherent cells [31]. FAK is an upstream regulator of the Akt signalling pathway in various cancer cell lines and xenograft tumour models to potentiate proliferation, migration and cell survival [46]. Focal adhesion kinase inactivation in non-adherent LAM/TSC cells leads to reduced activation of Akt, mTOR and S6. So the observed transient impairment of S6 phosphorylation might be related to the cell detachment, which is accompanied by the inhibition of FAK. The different status of S6 phosphorylation in adherent and non-adherent cells is consistent with the replication rate data, which is high in adherent LAM/TSC cells and very low in non-adherent cells. The impaired G1/S phase progression is a general feature of cells that have undergone EMT [47]. G1/S phase progression is reduced in non-adherent LAM/TSC cells, as it occurs for the expression of the markers of proliferation, suggesting that the reduced proliferation may favour the mobility of the cells in this condition. It was recently shown that the lack of plasma membrane E-cadherin in *Tsc2*^(−/−)^ mutant cells might be related to their reduced adhesive ability compared with the cells expressing tuberin, and this may lead to detachment and growth in suspension [30]. Epithelial-to-mesenchymal transition is an important event that endows cells with migratory and invasive properties inducing the invasive phenotype, and can represent the molecular base through which the TSC2 cells are able to migrate as proposed in the benign ‘metastatic’ theory of LAM [48]. The presence of mesenchymal tumours in the kidneys of patients affected by TSC suggests that hamartin and tuberin could regulate differentiation of an early renal precursor cell involved in TSC angiomyolipoma pathogenesis [49]. We observed that LAM/TSC cells display anchorage-independent growth and mesenchymal features. These EMT characteristics are dependent upon tuberin expression in LAM/TSC cells, as it had been shown in Tsc2^(−/−)^ Eker rat cell lines [30]. Epithelial-to-mesenchymal transition is accompanied by loss of cell–cell contacts, characterized by a down-regulation of E-cadherin expression [30]. Also, the inflammatory microenvironment may represent an important factor in the regulation of carcinoma cell migration from primary tumours through a process of EMT [20]. Likewise, IL-6 promotes EMT in breast cancer cells, and SNAIL can induce IL-6 expression [35,36]. Lymphangioleiomyomatosis/TSC cells secrete, probably in a tuberin-dependent manner, high amounts of IL-6 and IL-8 that cannot be significantly affected by the exposure to rapamycin or anti-EGFR antibodies. These data suggest that the mechanisms regulating interleukin production in LAM/TSC cells are dependent on the activity of pathways sensitive to tuberin.

The proliferation of LAM/TSC cells is EGF-dependent and the blockade of EGFR causes cell death as we had previously reported for TSC2^−/meth^ and TSC2^−/−^ ASM cells [16,19,27]. The action of the two used antibodies is ranging from immediate cell death to delayed cytotoxic/cytostatic reactions. Both anti-EGFR monoclonal antibodies prevent ligand binding and interrupt EGF signalling cascade; these data suggest that anti-EGFR antibody-Ab5 interaction may have some kind of early positive effects on LAM/TSC cells before becoming toxic. In LAM/TSC cells, rapamycin-mediated inhibition of mTOR causes the blocking of proliferation either when rapamycin was added at plating time or 3 hrs after plating; differently in TSC2^−/−^ and TSC2^−/meth^ ASM cells, rapamycin was efficacious only when added at plating time. The efficacy of rapamycin in LAM/TSC cells might be secondary to their specific ability to survive independently from the anchorage as demonstrated by the reduced viability of non-adherent cells after the drug incubation. These data confirmed the recent published findings, demonstrating the ability of Sirolimus to decrease the number of circulating LAM cells in blood and urine of LAM patients [50]. Together, all these data suggest that a valid pharmacological therapy for LAM/TSC cells has to control both proliferation and migration.

In conclusion, the current study confirmed that an epigenetic event may be rather common in LAM or TSC cells leading to impaired transcription of tuberin with a full deployment of TSC cellular phenotype. The chromatin-remodelling agents, triggering the tuberin transcription, can revert the mesenchymal features such as E-cadherin expression and block interleukins secretion. In addition, the LAM/TSC cells display the ability to survive independently from anchorage acquiring invasive characteristics. Rapamycin and anti-EGFR antibodies are capable of affecting the survival of non-adherent LAM cells.

## Supporting information

**Figure S1** Densitometric analysis of protein levels of phospho-S6, S6, phospho-Erk, Erk was evaluated by western blotting (Fig. 3B) following incubation with rapamycin and EGFR Ab3 for 6 days 3 hrs after plating. Densitometric analysis was evaluated relatively to β-actin levels. Error bars represent the SD for four independent experiments. **P* < 0.05, ***P* < 0.01, ****P* < 0.001 *versus* control (anova with Bonferroni*s test).

**Figure S2** Densitometric analysis of protein levels of phospho-FAK, phospho-Akt, phospho-mTOR (Ser 2448), phospho-mTOR (Ser2481) and phospho-S6 was evaluated by western blotting (Fig. 4G) in A and NA LAM/TSC cells and after incubation with PF228 (1 or 10 μM). Densitometric analysis was measured relatively to β-actin levels. Error bars represent the SD for four independent experiments. **P* < 0.05, ***P* < 0.01, ****P* < 0.001 *versus* A cells (anova with Bonferroni*s test).

**Figure S3** (**A**) Levels of phospho-FAK, phospho-Akt, phospho-mTOR (Ser 2448), phospho-mTOR (Ser2481) and phospho-S6 (representative experiments) were evaluated by western blotting in TSC2^−/meth^, TSC2^−/−^ ASM cells and in HASMCs after incubation with PF573228 (PF228; 1 or 10 μM). β-actin was evaluated as loading control. (**B**) Relative intensity by densitometric analysis was measured relatively to β-actin levels. Error bars represent the SD for four independent experiments. **P* < 0.05, ***P* < 0.01, ****P* < 0.001 *versus* control of each group (anova with Bonferroni*s test).
