# Supplementary figures and images for: TSC2 epigenetic defect in primary LAM cells. Evidence of an anchorage-independent survival
Source: J Cell Mol Med. 2014 Mar 7;18(5):766–79. doi: 10.1111/jcmm.12237 (PMC4119383; doi:10.1111/jcmm.12237)

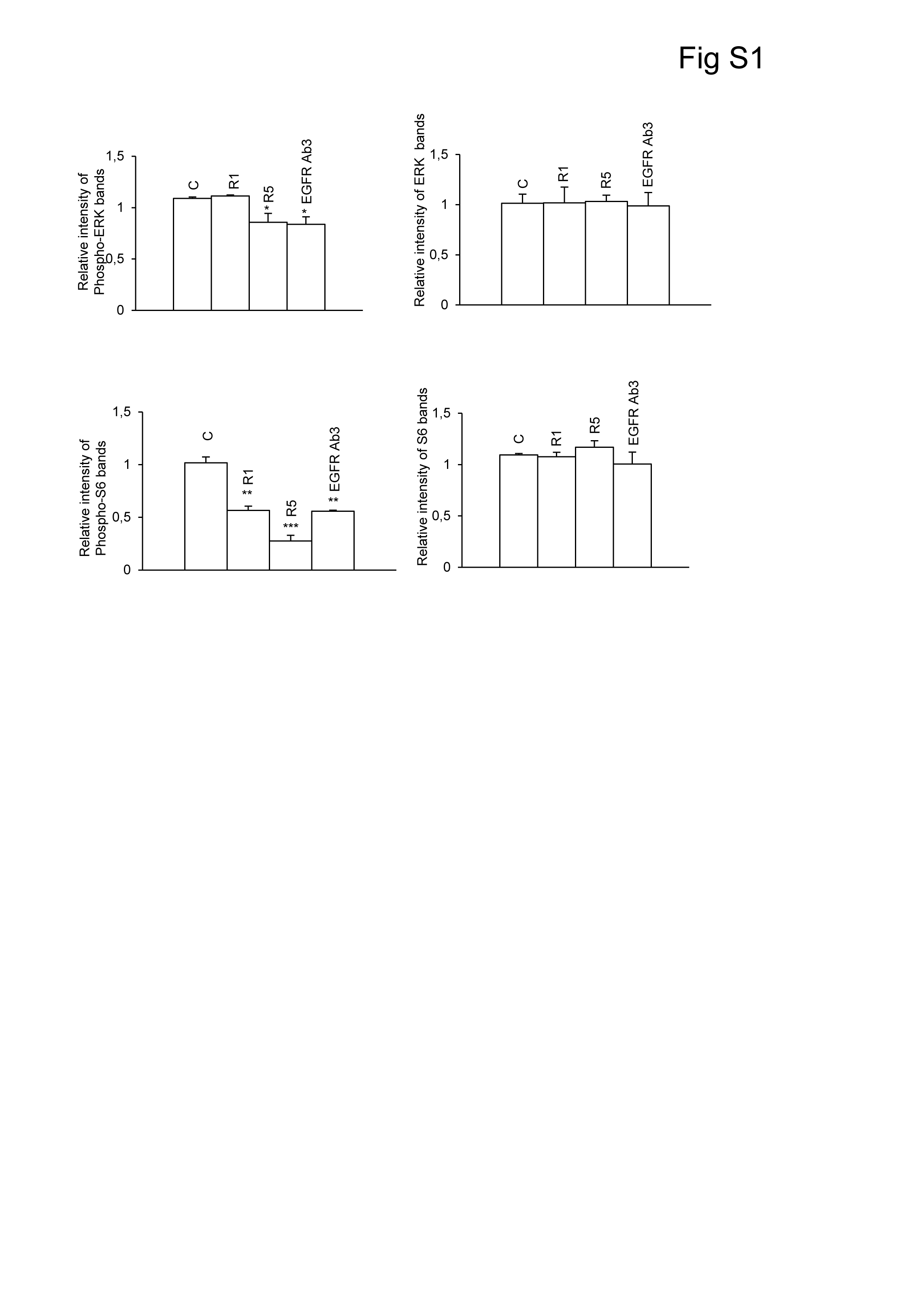

Supplement: Supplementary file 1 — Figure S1 Densitometric analysis of protein levels of phospho-S6, S6, phospho-Erk, Erk was evaluated by western blotting (Fig. 3B) following incubation with rapamycin and EGFR Ab3 for 6 days 3 hrs after plating. Densitometric analysis was evaluated relatively to β-actin levels. Error bars represent the SD for four independent experiments. *P < 0.05, **P < 0.01, ***P < 0.001 versus control (anova with Bonferroni*s test). [file jcmm0018-0766-SD1.tif]

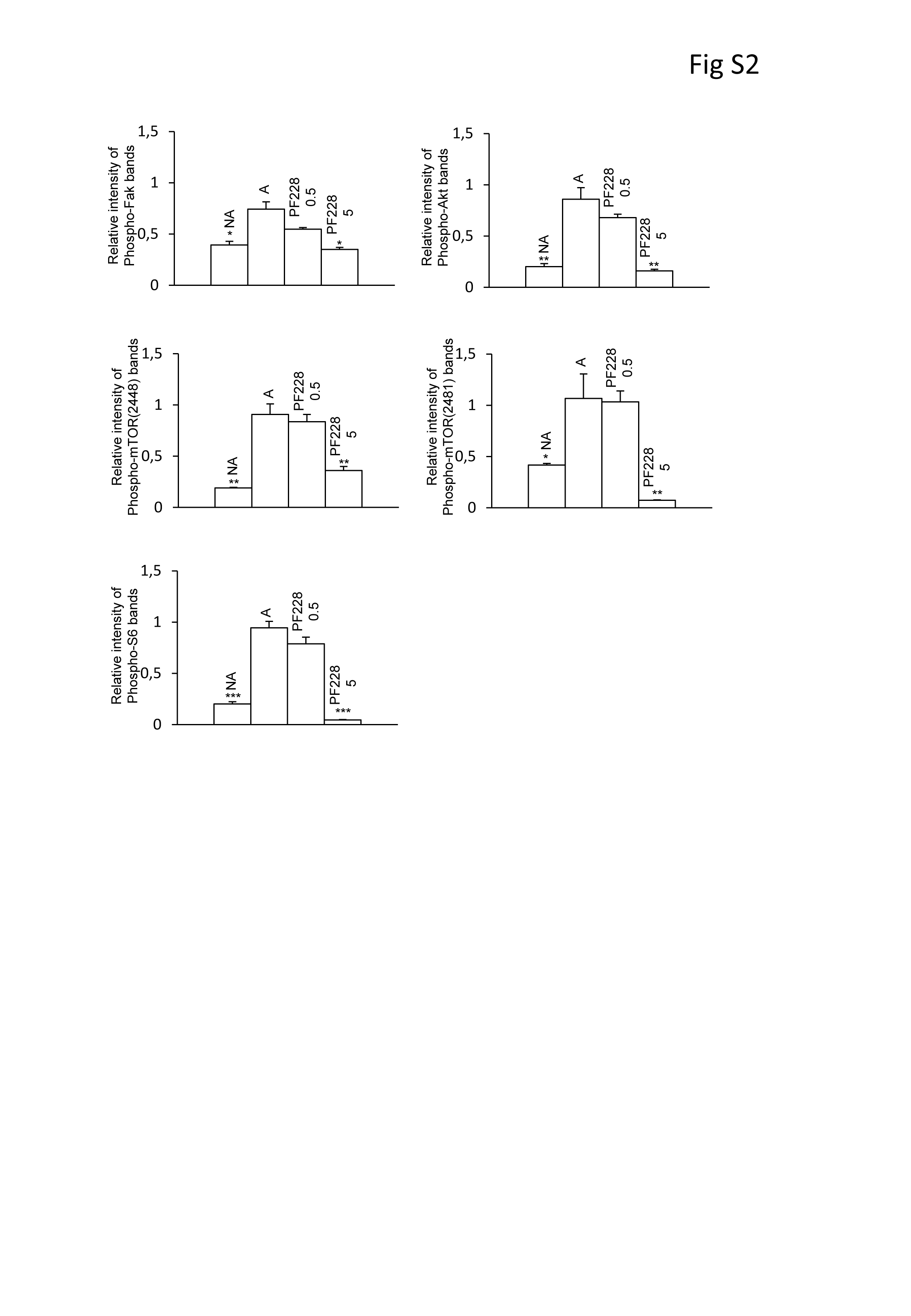

Supplement: Supplementary file 2 — Figure S2 Densitometric analysis of protein levels of phospho-FAK, phospho-Akt, phospho-mTOR (Ser 2448), phospho-mTOR (Ser2481) and phospho-S6 was evaluated by western blotting (Fig. 4G) in A and NA LAM/TSC cells and after incubation with PF228 (1 or 10 μM). Densitometric analysis was measured relatively to β-actin levels. Error bars represent the SD for four independent experiments. *P < 0.05, **P < 0.01, ***P < 0.001 versus A cells (anova with Bonferroni*s test). [file jcmm0018-0766-SD2.tif]

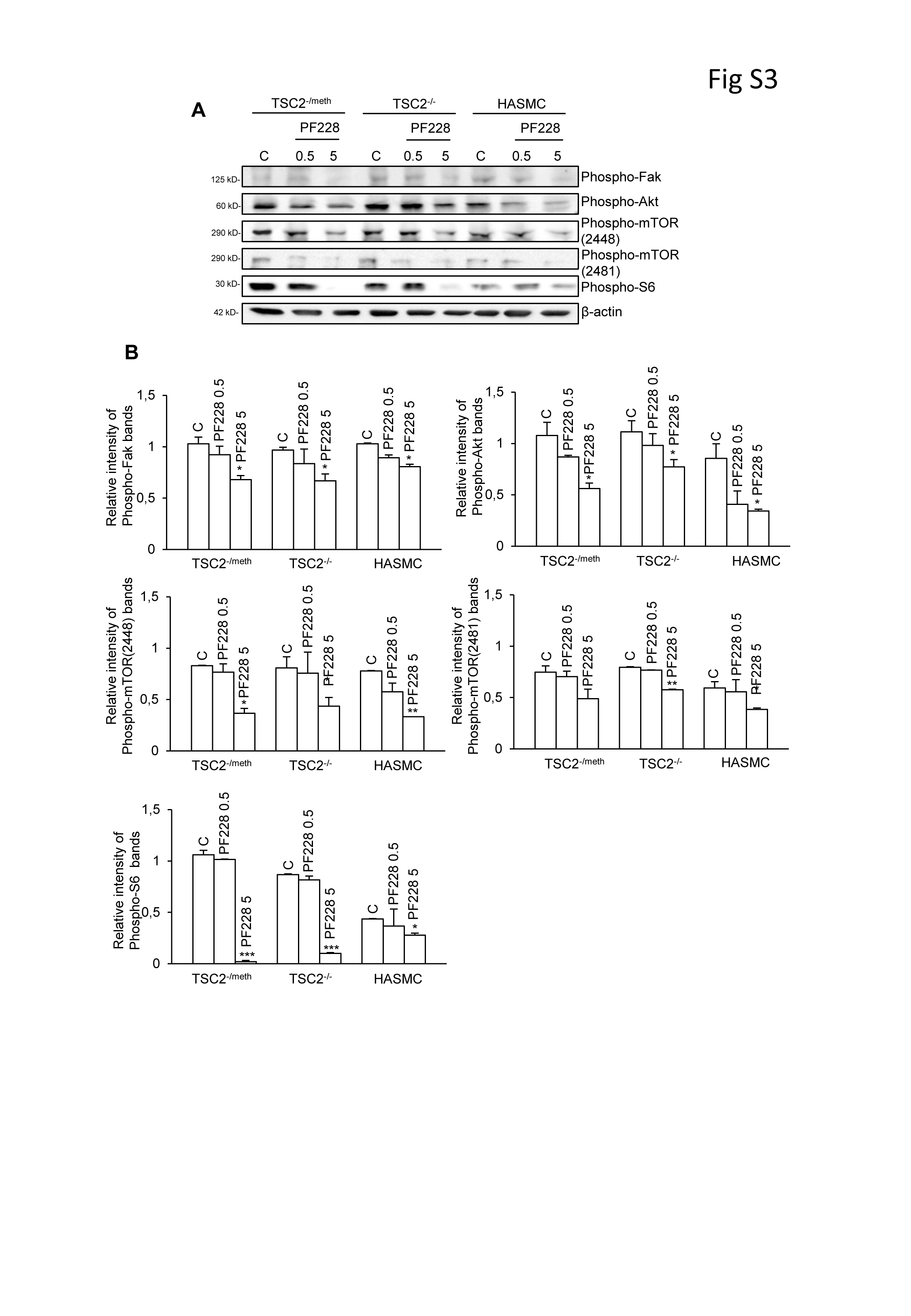

Supplement: Supplementary file 3 — Figure S3 (A) Levels of phospho-FAK, phospho-Akt, phospho-mTOR (Ser 2448), phospho-mTOR (Ser2481) and phospho-S6 (representative experiments) were evaluated by western blotting in TSC2−/meth, TSC2−/− ASM cells and in HASMCs after incubation with PF573228 (PF228; 1 or 10 μM). β-actin was evaluated as loading control. (B) Relative intensity by densitometric analysis was measured relatively to β-actin levels. Error bars represent the SD for four independent experiments. *P < 0.05, **P < 0.01, ***P < 0.001 versus control of each group (anova with Bonferroni*s test). [file jcmm0018-0766-SD3.tif]
